# Supplementary figures and images for: Exploration of gray matter alterations and cognitive function impairment in adolescents with first-episode non-suicidal self-injury and the associations with self-injury characteristics
Source: PeerJ. 2025 Aug 26;13:e19914. doi: 10.7717/peerj.19914 (PMC12396207; doi:10.7717/peerj.19914)

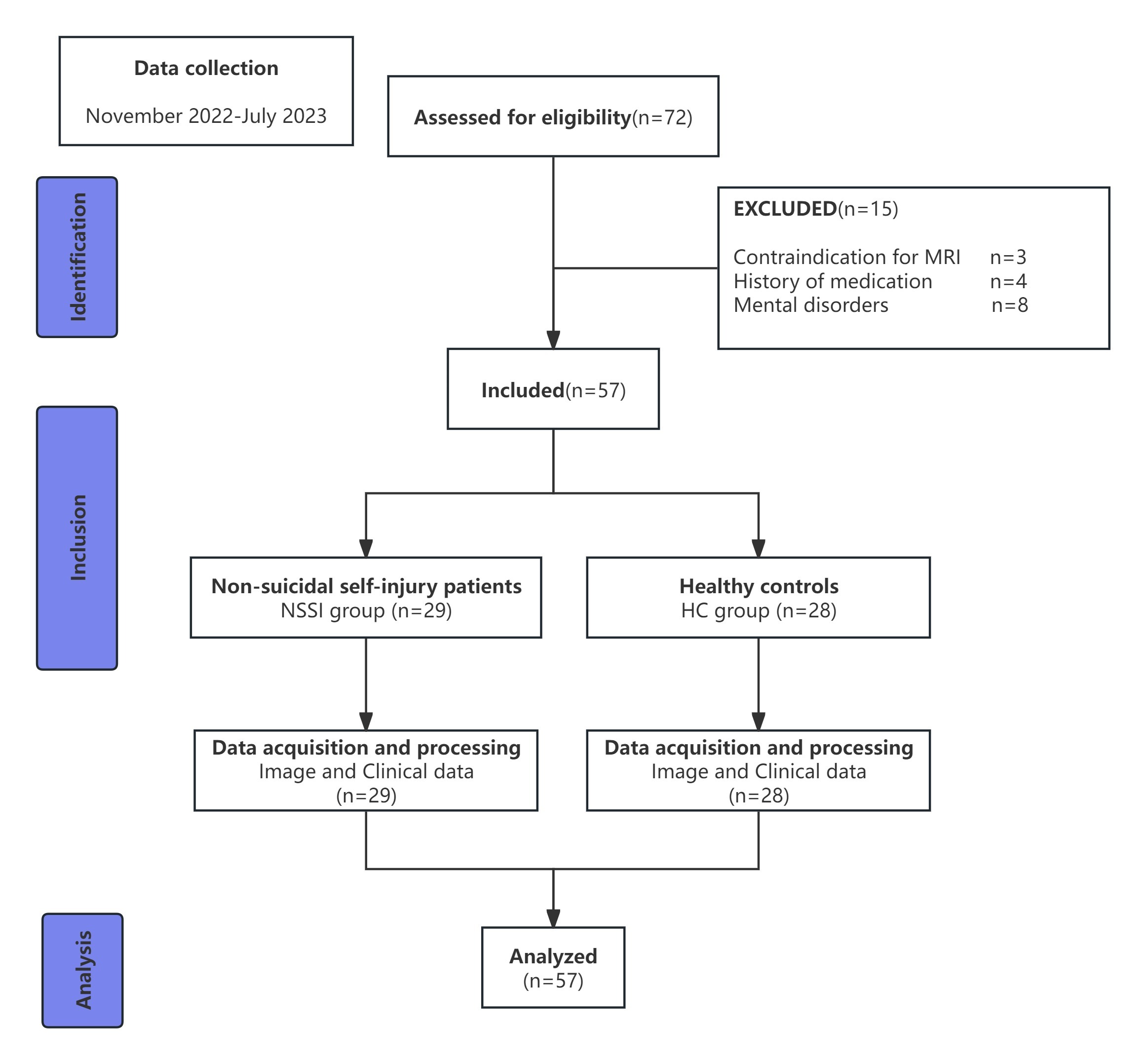

Supplement: Supplemental Information 3 [file peerj-13-19914-s003.jpg]
