# Supplementary material for: Exploration of gray matter alterations and cognitive function impairment in adolescents with first-episode non-suicidal self-injury and the associations with self-injury characteristics
Source: PeerJ. 2025 Aug 26;13:e19914. doi: 10.7717/peerj.19914 (PMC12396207; doi:10.7717/peerj.19914)
Supplement: Supplemental Information 4 — NSSI, non-suicidal self-injury; HC, healthy control. [file peerj-13-19914-s004.docx]

**Subcortical volume differences between NSSI group and HC group**

| Region | NSSI(mm^3^) | HC(mm^3^) | *F* statistic | FDR Corrected  *q* statistic |
| --- | --- | --- | --- | --- |
| Left Thalamus | 8129.393±787.723 | 8366.989±873.280 | 0.183 | 0.761 |
| Left Caudate | 3684.203±451.130 | 3688.021±555.153 | 1.125 | 0.515 |
| Left Putamen | 4880.397±410.755 | 5310.075±525.930 | 7.558 | **0.028** |
| Left Pallidum | 2007.100±212.426 | 2081.390±199.876 | 0.557 | 0.643 |
| Left Hippocampus | 4005.648±269.676 | 4138.389±230.609 | 1.633 | 0.483 |
| Left Amygdala | 1452.852±167.240 | 1466.779±146.579 | 0.093 | 0.761 |
| Left Accumbens | 285.883±56.799 | 338.129±63.728 | 8.179 | **0.028** |
| Right Thalamus | 7379.166±587.617 | 7533.504±735.138 | 0.005 | 0.941 |
| Right Caudate | 3707.186±501.078 | 3853.218±432.136 | 0.250 | 0.722 |
| Right Putamen | 5385.086±558.370 | 5806.564±569.662 | 5.338 | 0.175 |
| Right Pallidum | 2057.538±216.327 | 2158.261±185.210 | 2.068 | 0.474 |
| Right Hippocampus | 4249.438±292.637 | 4354.871±274.569 | 0.638 | 0.599 |
| Right Amygdala | 1684.697±146.926 | 1702.293±178.698 | 0.866 | 0.599 |
| Right Accumbens | 456.434±73.572 | 479.821±60.706 | 1.665 | 0.474 |

**Cortical volume differences between NSSI group and HC group**

| Region | NSSI(mm^3^) | HC(mm^3^) | *F* statistic | FDR Corrected  *q* statistic |
| --- | --- | --- | --- | --- |
| Left bankssts | 2479.590±401.720 | 2460.180±434.270 | 0.012 | 0.996 |
| Left caudal anterior cingulate | 1733.340±629.282 | 1476.750±573.089 | 2.047 | 0.581 |
| Left caudal middle frontal | 6991.410±1127.734 | 7278.640±1256.284 | 0.066 | 0.925 |
| Left cuneus | 2961.970±495.742 | 2955.460±485.320 | 0.309 | 0.801 |
| Left entorhinal | 2003.860±447.888 | 1961.930±353.459 | 1.684 | 0.597 |
| Left fusiform | 9675.100±1089.810 | 9873.390±1072.347 | <0.001 | 0.996 |
| Left inferior parietal | 13205.520±1930.723 | 13208.610±1587.779 | <0.001 | 0.996 |
| Left inferior temporal | 11693.760±1855.546 | 12050.820±1696.774 | 0.055 | 0.925 |
| Left isthmus cingulate | 2758.760±420.508 | 3010.040±362.222 | 5.081 | 0.329 |
| Left lateral occipital | 11745.100±1385.233 | 11488.540±1638.078 | 4.159 | 0.399 |
| Left lateral orbitofrontal | 7966.280±699.094 | 8067.570±897.289 | 0.297 | 0.801 |
| Left lingual | 5834.140±834.618 | 6217.070±961.063 | 1.132 | 0.662 |
| Left medial orbitofrontal | 5786.000±578.940 | 5592.680±724.023 | 6.118 | 0.289 |
| Left middle temporal | 10453.690±1519.207 | 11317.040±1104.302 | 3.392 | 0.453 |
| Left parahippocampal | 2041.280±294.460 | 2004.820±237.961 | 0.203 | 0.845 |
| Left paracentral | 3578.140±401.226 | 3621.430±481.301 | 0.545 | 0.751 |
| Left pars opercularis | 4690.070±532.984 | 5117.540±1186.232 | 0.809 | 0.729 |
| Left pars orbitalis | 2704.030±279.327 | 2709.460±391.356 | 0.182 | 0.845 |
| Left pars triangularis | 4198.620±501.068 | 4101.610±657.691 | 2.292 | 0.578 |
| Left pericalcarine | 1977.000±342.482 | 2101.540±474.330 | 0.436 | 0.791 |
| Left postcentral | 9856.760±1008.770 | 10025.180±1415.415 | 0.003 | 0.996 |
| Left posterior cingulate | 3119.720±632.232 | 3337.860±624.005 | 0.831 | 0.729 |
| Left precentral | 13792.480±1271.415 | 13978.110±1479.878 | 0.635 | 0.729 |
| Left precuneus | 10552.620±1115.998 | 10974.000±1319.420 | 0.685 | 0.729 |
| Left rostral anterior cingulate | 2693.790±584.117 | 2299.570±636.780 | 12.368 | **0.034** |
| Left rostral middle frontal | 17189.720±2045.871 | 17404.680±2112.901 | 0.700 | 0.729 |
| Left superior frontal | 25267.620±2083.631 | 26448.960±2631.003 | 1.925 | 0.581 |
| Left superior parietal | 14040.590±1581.332 | 14721.710±1605.437 | 1.379 | 0.597 |
| Left superior temporal | 12771.900±1629.996 | 12592.640±2158.270 | 1.404 | 0.597 |
| Left supramarginal | 12060.480±1980.906 | 12300.000±2015.097 | 0.295 | 0.801 |
| Left frontal pole | 1209.970±182.656 | 1152.680±164.995 | 2.342 | 0.578 |
| Left temporal pole | 2731.340±385.160 | 2737.960±562.163 | 0.147 | 0.854 |
| Left transverse temporal | 1280.480±222.239 | 1214.140±361.537 | 3.187 | 0.453 |
| Left insula | 7304.860±661.099 | 7306.930±808.151 | 1.557 | 0.597 |
| Right bankssts | 2356.900±332.587 | 2357.640±359.496 | 0.010 | 0.983 |
| Right caudal anterior cingulate | 2030.620±653.107 | 2163.570±719.537 | 0.003 | 0.983 |
| Right caudal middle frontal | 6299.310±1143.917 | 6734.610±1182.399 | 0.563 | 0.863 |
| Right cuneus | 3068.900±397.327 | 3137.320±517.024 | 0.233 | 0.863 |
| Right entorhinal | 1971.140±421.504 | 1971.710±334.490 | 0.294 | 0.863 |
| Right fusiform | 9457.240±1129.412 | 9283.820±1032.591 | 2.652 | 0.863 |
| Right inferior parietal | 16217.590±1910.567 | 16150.000±1692.754 | 2.597 | 0.863 |
| Right inferior temporal | 11813.070±1305.536 | 11745.360±1926.032 | 1.869 | 0.863 |
| Right isthmus cingulate | 2617.280±456.145 | 2761.540±508.377 | 0.480 | 0.863 |
| Right lateral occipital | 11804.620±1252.903 | 11726.610±1608.287 | 1.209 | 0.863 |
| Right lateral orbitofrontal | 8036.520±786.919 | 8170.250±914.442 | 0.046 | 0.983 |
| Right lingual | 6071.480±934.427 | 6652.430±1078.682 | 2.156 | 0.863 |
| Right medial orbitofrontal | 5870.340±607.738 | 5932.860±786.137 | 1.047 | 0.863 |
| Right middle temporal | 12375.100±1402.382 | 12531.180±1261.666 | 0.009 | 0.983 |
| Right parahippocampal | 1907.790±233.132 | 1880.860±236.968 | 0.166 | 0.863 |
| Right paracentral | 3826.280±601.138 | 4153.460±547.960 | 3.337 | 0.863 |
| Right pars opercularis | 4186.860±591.625 | 4240.750±836.444 | 0.245 | 0.863 |
| Right pars orbitalis | 3104.070±282.420 | 3066.250±418.346 | 0.299 | 0.863 |
| Right pars triangularis | 4879.690±713.340 | 4695.960±851.158 | 1.260 | 0.863 |
| Right pericalcarine | 2084.860±290.842 | 2330.640±413.770 | 3.150 | 0.863 |
| Right postcentral | 9332.030±1191.745 | 9643.290±1360.952 | 0.216 | 0.863 |
| Right posterior cingulate | 3259.590±971.724 | 3268.710±552.122 | <0.001 | 0.983 |
| Right precentral | 13461.000±1348.194 | 13567.640±1845.296 | 0.337 | 0.863 |
| Right precuneus | 11028.590±1219.580 | 11115.390±1195.666 | 0.639 | 0.863 |
| Right rostral anterior cingulate | 1942.070±519.218 | 1978.930±404.028 | 0.001 | 0.983 |
| Right rostral middle frontal | 17483.930±1765.610 | 17596.000±2773.985 | 0.333 | 0.863 |
| Right superior frontal | 23493.480±2199.725 | 24726.210±2826.480 | 1.782 | 0.863 |
| Right superior parietal | 13878.790±1452.056 | 13961.180±1983.487 | 0.703 | 0.863 |
| Right superior temporal | 11692.240±1306.944 | 11750.000±1164.377 | 0.245 | 0.863 |
| Right supramarginal | 10300.970±1168.414 | 10399.890±1413.955 | 0.309 | 0.863 |
| Right frontal pole | 1556.140±242.331 | 1517.890±181.889 | 1.260 | 0.863 |
| Right temporal pole | 2833.480±456.979 | 2919.540±661.364 | 0.015 | 0.983 |
| Right transverse temporal | 984.070±159.773 | 961.710±138.500 | 0.864 | 0.863 |
| Right insula | 7161.790±676.874 | 7321.250±918.331 | 0.173 | 0.863 |

**Cortical surface area differences between NSSI group and HC group**

| Region | NSSI(mm^2^) | HC(mm^2^) | *F* statistic | FDR Corrected  *q* statistic |
| --- | --- | --- | --- | --- |
| Left bankssts | 1034.140±140.068 | 1036.360±188.890 | 0.013 | 0.997 |
| Left caudal anterior cingulate | 546.000±133.626 | 493.430±135.768 | 3.896 | 0.388 |
| Left caudal middle frontal | 2284.690±298.202 | 2372.460±396.788 | <0.001 | 0.997 |
| Left cuneus | 1488.100±229.752 | 1474.500±222.801 | 1.347 | 0.711 |
| Left entorhinal | 413.690±91.364 | 432.110±63.936 | 0.045 | 0.997 |
| Left fusiform | 2957.070±266.086 | 3048.820±322.364 | 0.052 | 0.997 |
| Left inferior parietal | 4674.170±662.714 | 4726.820±577.378 | 0.065 | 0.997 |
| Left inferior temporal | 3467.830±492.825 | 3476.110±490.307 | 1.115 | 0.774 |
| Left isthmus cingulate | 935.790±155.473 | 1052.960±214.210 | 5.451 | 0.388 |
| Left lateral occipital | 5051.830±563.959 | 4990.750±722.415 | 2.391 | 0.544 |
| Left lateral orbitofrontal | 2244.210±231.092 | 2231.710±297.309 | 2.599 | 0.544 |
| Left lingual | 2938.340±296.332 | 2995.890±409.175 | 0.008 | 0.997 |
| Left medial orbitofrontal | 1892.860±223.120 | 1841.320±222.696 | 3.796 | 0.388 |
| Left middle temporal | 3253.340±410.089 | 3387.680±427.177 | 0.006 | 0.997 |
| Left parahippocampal | 632.760±64.560 | 663.790±68.391 | 1.762 | 0.587 |
| Left paracentral | 1400.170±150.188 | 1444.040±210.668 | 0.031 | 0.997 |
| Left pars opercularis | 1539.000±189.476 | 1656.460±350.218 | 0.354 | 0.858 |
| Left pars orbitalis | 630.000±76.647 | 653.250±101.238 | 0.040 | 0.997 |
| Left pars triangularis | 1320.210±186.792 | 1327.890±229.530 | 0.877 | 0.786 |
| Left pericalcarine | 1328.070±170.878 | 1409.140±250.649 | 0.444 | 0.858 |
| Left postcentral | 4161.930±359.861 | 4265.320±499.259 | 0.004 | 0.997 |
| Left posterior cingulate | 1114.830±247.298 | 1152.860±205.703 | <0.001 | 0.997 |
| Left precentral | 4789.760±349.678 | 4913.820±539.349 | 0.487 | 0.858 |
| Left precuneus | 3807.930±407.588 | 3992.930±579.928 | 0.367 | 0.858 |
| Left rostral anterior cingulate | 753.100±167.061 | 687.110±169.974 | 7.255 | 0.340 |
| Left rostral middle frontal | 5659.140±717.433 | 5697.710±795.963 | 2.126 | 0.570 |
| Left superior frontal | 7199.000±564.313 | 7578.860±913.644 | 1.011 | 0.775 |
| Left superior parietal | 5459.070±596.361 | 5740.180±729.918 | 0.820 | 0.786 |
| Left superior temporal | 4072.660±472.417 | 4045.610±698.688 | 1.904 | 0.587 |
| Left supramarginal | 4276.280±727.766 | 4375.500±795.136 | 0.427 | 0.858 |
| Left frontal pole | 242.410±29.239 | 245.710±22.132 | 0.406 | 0.858 |
| Left temporal pole | 453.480±57.453 | 467.460±71.056 | 0.151 | 0.997 |
| Left transverse temporal | 483.210±65.551 | 467.180±145.253 | 2.878 | 0.544 |
| Left insula | 2271.280±232.131 | 2235.540±252.008 | 4.179 | 0.388 |
| Right bankssts | 972.900±130.598 | 971.390±139.951 | 0.050 | 0.985 |
| Right caudal anterior cingulate | 641.170±172.987 | 672.960±178.087 | 0.003 | 0.985 |
| Right caudal middle frontal | 2049.340±333.123 | 2195.250±375.179 | 0.733 | 0.902 |
| Right cuneus | 1508.930±201.700 | 1569.890±237.359 | 0.004 | 0.985 |
| Right entorhinal | 363.480±55.408 | 379.500±71.681 | 0.015 | 0.985 |
| Right fusiform | 2997.450±278.651 | 2976.610±354.710 | 2.798 | 0.902 |
| Right inferior parietal | 5590.550±734.140 | 5677.320±693.707 | 1.195 | 0.902 |
| Right inferior temporal | 3494.030±391.515 | 3530.390±588.240 | 1.078 | 0.902 |
| Right isthmus cingulate | 870.170±135.199 | 892.000±124.117 | 0.040 | 0.985 |
| Right lateral occipital | 4938.930±568.177 | 4886.890±557.311 | 1.628 | 0.902 |
| Right lateral orbitofrontal | 2311.660±243.671 | 2417.320±275.711 | 1.040 | 0.902 |
| Right lingual | 3038.450±377.912 | 3213.040±480.066 | 0.370 | 0.975 |
| Right medial orbitofrontal | 1855.520±198.038 | 1926.290±249.322 | 0.032 | 0.985 |
| Right middle temporal | 3745.690±434.455 | 3746.430±475.498 | 1.324 | 0.902 |
| Right parahippocampal | 611.900±63.760 | 637.070±93.587 | 0.765 | 0.902 |
| Right paracentral | 1482.000±194.045 | 1561.500±210.172 | 0.858 | 0.902 |
| Right pars opercularis | 1369.450±181.530 | 1408.250±270.181 | 0.171 | 0.985 |
| Right pars orbitalis | 761.660±93.780 | 757.250±84.036 | 0.420 | 0.975 |
| Right pars triangularis | 1574.410±245.533 | 1554.430±252.014 | 0.728 | 0.902 |
| Right pericalcarine | 1457.170±191.694 | 1588.430±264.216 | 1.474 | 0.902 |
| Right postcentral | 3976.000±421.340 | 4226.610±551.054 | 1.989 | 0.902 |
| Right posterior cingulate | 1125.620±240.220 | 1121.540±151.605 | 0.142 | 0.985 |
| Right precentral | 4707.380±443.868 | 4937.320±545.083 | 1.437 | 0.902 |
| Right precuneus | 3954.170±459.694 | 4039.180±591.257 | 0.307 | 0.985 |
| Right rostral anterior cingulate | 542.520±160.577 | 562.320±87.439 | 0.025 | 0.985 |
| Right rostral middle frontal | 5665.380±545.007 | 5736.210±848.550 | 0.561 | 0.914 |
| Right superior frontal | 6690.790±588.503 | 7056.460±947.603 | 0.639 | 0.910 |
| Right superior parietal | 5375.690±585.648 | 5503.820±786.788 | 0.127 | 0.985 |
| Right superior temporal | 3702.790±346.921 | 3830.000±374.074 | 0.226 | 0.985 |
| Right supramarginal | 3807.860±469.486 | 3933.820±598.082 | 0.081 | 0.985 |
| Right frontal pole | 307.760±32.773 | 301.110±30.496 | 3.901 | 0.902 |
| Right temporal pole | 446.480±61.691 | 482.960±91.836 | 0.744 | 0.902 |
| Right transverse temporal | 340.410±44.082 | 345.290±42.197 | <0.001 | 0.997 |
| Right insula | 2208.660±243.894 | 2290.540±298.538 | 0.023 | 0.985 |

**Cortical thickness differences between NSSI group and HC group**

| Region | NSSI(mm) | HC(mm) | *F* statistic | FDR Corrected  *q* statistic |
| --- | --- | --- | --- | --- |
| Left bankssts | 2.512±0.177 | 2.480±0.186 | 0.062 | 0.969 |
| Left caudal anterior cingulate | 2.813±0.289 | 2.750±0.315 | 0.058 | 0.969 |
| Left caudal middle frontal | 2.778±0.098 | 2.807±0.140 | 2.028 | 0.646 |
| Left cuneus | 1.890±0.132 | 1.896±0.202 | 0.267 | 0.969 |
| Left entorhinal | 3.497±0.240 | 3.372±0.353 | 1.976 | 0.646 |
| Left fusiform | 2.846±0.105 | 2.845±0.130 | 0.013 | 0.990 |
| Left inferior parietal | 2.557±0.111 | 2.561±0.144 | 0.218 | 0.969 |
| Left inferior temporal | 2.848±0.144 | 2.921±0.142 | 2.238 | 0.646 |
| Left isthmus cingulate | 2.585±0.246 | 2.576±0.245 | <0.001 | 0.990 |
| Left lateral occipital | 2.155±0.085 | 2.143±0.091 | 0.421 | 0.969 |
| Left lateral orbitofrontal | 3.068±0.149 | 3.048±0.185 | 0.005 | 0.990 |
| Left lingual | 1.933±0.125 | 1.990±0.141 | 2.556 | 0.646 |
| Left medial orbitofrontal | 2.620±0.236 | 2.560±0.150 | 1.596 | 0.721 |
| Left middle temporal | 2.745±0.205 | 2.801±0.172 | 2.175 | 0.646 |
| Left parahippocampal | 2.857±0.297 | 2.697±0.282 | 2.410 | 0.646 |
| Left paracentral | 2.439±0.105 | 2.412±0.156 | 0.250 | 0.969 |
| Left pars opercularis | 2.671±0.130 | 2.681±0.173 | 0.173 | 0.969 |
| Left pars orbitalis | 3.008±0.195 | 2.980±0.199 | 0.001 | 0.990 |
| Left pars triangularis | 2.642±0.123 | 2.610±0.163 | 0.137 | 0.969 |
| Left pericalcarine | 1.639±0.129 | 1.633±0.144 | 0.043 | 0.969 |
| Left postcentral | 2.149±0.094 | 2.126±0.106 | 0.406 | 0.969 |
| Left posterior cingulate | 2.583±0.256 | 2.649±0.324 | 1.386 | 0.757 |
| Left precentral | 2.655±0.122 | 2.634±0.119 | 0.118 | 0.969 |
| Left precuneus | 2.554±0.122 | 2.562±0.154 | 0.820 | 0.969 |
| Left rostral anterior cingulate | 3.016±0.248 | 2.908±0.244 | 2.448 | 0.646 |
| Left rostral middle frontal | 2.573±0.116 | 2.570±0.130 | 0.135 | 0.969 |
| Left superior frontal | 3.016±0.098 | 3.030±0.148 | 1.927 | 0.646 |
| Left superior parietal | 2.311±0.087 | 2.311±0.132 | 0.357 | 0.969 |
| Left superior temporal | 2.741±0.147 | 2.738±0.145 | 0.034 | 0.969 |
| Left supramarginal | 2.593±0.143 | 2.599±0.145 | 0.223 | 0.969 |
| Left frontal pole | 3.143±0.318 | 2.951±0.295 | 3.965 | 0.646 |
| Left temporal pole | 3.759±0.318 | 3.717±0.325 | 0.317 | 0.969 |
| Left transverse temporal | 2.492±0.170 | 2.480±0.214 | 0.068 | 0.969 |
| Left insula | 3.217±0.127 | 3.236±0.152 | 0.306 | 0.969 |
| Right bankssts | 2.565±0.195 | 2.576±0.192 | 0.467 | 0.770 |
| Right caudal anterior cingulate | 2.607±0.310 | 2.656±0.279 | 0.226 | 0.792 |
| Right caudal middle frontal | 2.735±0.104 | 2.754±0.141 | 0.854 | 0.748 |
| Right cuneus | 1.907±0.105 | 1.869±0.137 | 0.776 | 0.748 |
| Right entorhinal | 3.661±0.299 | 3.557±0.302 | 0.732 | 0.748 |
| Right fusiform | 2.826±0.146 | 2.795±0.123 | 0.194 | 0.792 |
| Right inferior parietal | 2.559±0.105 | 2.539±0.136 | 0.016 | 0.956 |
| Right inferior temporal | 2.862±0.151 | 2.842±0.138 | 0.157 | 0.792 |
| Right isthmus cingulate | 2.549±0.277 | 2.625±0.252 | 1.054 | 0.748 |
| Right lateral occipital | 2.229±0.123 | 2.226±0.139 | 0.001 | 0.979 |
| Right lateral orbitofrontal | 3.026±0.149 | 2.970±0.151 | 0.851 | 0.748 |
| Right lingual | 1.940±0.104 | 1.999±0.124 | 4.469 | 0.442 |
| Right medial orbitofrontal | 2.662±0.201 | 2.599±0.164 | 1.624 | 0.748 |
| Right middle temporal | 2.813±0.176 | 2.843±0.232 | 1.186 | 0.748 |
| Right parahippocampal | 2.784±0.232 | 2.669±0.281 | 1.582 | 0.748 |
| Right paracentral | 2.487±0.118 | 2.535±0.151 | 2.228 | 0.748 |
| Right pars opercularis | 2.711±0.130 | 2.664±0.141 | 1.288 | 0.748 |
| Right pars orbitalis | 3.027±0.167 | 3.034±0.249 | 0.204 | 0.792 |
| Right pars triangularis | 2.655±0.129 | 2.591±0.150 | 1.529 | 0.748 |
| Right pericalcarine | 1.584±0.126 | 1.618±0.127 | 1.851 | 0.748 |
| Right postcentral | 2.117±0.087 | 2.074±0.151 | 0.827 | 0.748 |
| Right posterior cingulate | 2.573±0.390 | 2.592±0.321 | 0.134 | 0.792 |
| Right precentral | 2.632±0.080 | 2.557±0.145 | 5.164 | 0.442 |
| Right precuneus | 2.566±0.093 | 2.574±0.170 | 0.468 | 0.770 |
| Right rostral anterior cingulate | 2.881±0.300 | 2.903±0.306 | 0.187 | 0.792 |
| Right rostral middle frontal | 2.589±0.128 | 2.562±0.123 | 0.293 | 0.792 |
| Right superior frontal | 3.003±0.095 | 3.008±0.134 | 1.356 | 0.748 |
| Right superior parietal | 2.308±0.084 | 2.293±0.118 | 0.003 | 0.979 |
| Right superior temporal | 2.752±0.134 | 2.707±0.147 | 0.854 | 0.748 |
| Right supramarginal | 2.532±0.146 | 2.499±0.101 | 0.625 | 0.770 |
| Right frontal pole | 3.170±0.297 | 3.129±0.282 | 0.128 | 0.792 |
| Right temporal pole | 3.882±0.367 | 3.841±0.277 | 0.158 | 0.792 |
| Right transverse temporal | 2.704±0.192 | 2.583±0.160 | 6.104 | 0.442 |
| Right insula | 3.244±0.160 | 3.181±0.146 | 0.551 | 0.770 |
